# Depth-dependent geochemical and microbiological gradients in Fe(III) deposits resulting from coal mine-derived acid mine drainage

**DOI:** 10.3389/fmicb.2014.00215

**Published:** 2014-05-14

**Authors:** Justin S. Brantner, Zachary J. Haake, John E. Burwick, Christopher M. Menge, Shane T. Hotchkiss, John M. Senko

**Affiliations:** ^1^Department of Biology, The University of AkronAkron, OH, USA; ^2^Integrated Bioscience Program, The University of AkronAkron, OH, USA; ^3^Department of Geosciences, The University of AkronAkron, OH, USA; ^4^Department of Chemistry, The University of AkronAkron, OH, USA

**Keywords:** acid mine drainage, Fe(II) oxidizing bacteria, Fe(III) reducing bacteria

## Abstract

We evaluated the depth-dependent geochemistry and microbiology of sediments that have developed via the microbially-mediated oxidation of Fe(II) dissolved in acid mine drainage (AMD), giving rise to a 8–10 cm deep “iron mound” that is composed primarily of Fe(III) (hydr)oxide phases. Chemical analyses of iron mound sediments indicated a zone of maximal Fe(III) reducing bacterial activity at a depth of approximately 2.5 cm despite the availability of dissolved O_2_ at this depth. Subsequently, Fe(II) was depleted at depths within the iron mound sediments that did not contain abundant O_2_. Evaluations of microbial communities at 1 cm depth intervals within the iron mound sediments using “next generation” nucleic acid sequencing approaches revealed an abundance of phylotypes attributable to acidophilic Fe(II) oxidizing Betaproteobacteria and the chloroplasts of photosynthetic microeukaryotic organisms in the upper 4 cm of the iron mound sediments. While we observed a depth-dependent transition in microbial community structure within the iron mound sediments, phylotypes attributable to Gammaproteobacterial lineages capable of both Fe(II) oxidation and Fe(III) reduction were abundant in sequence libraries (comprising ≥20% of sequences) from all depths. Similarly, abundances of total cells and culturable Fe(II) oxidizing bacteria were uniform throughout the iron mound sediments. Our results indicate that O_2_ and Fe(III) reduction co-occur in AMD-induced iron mound sediments, but that Fe(II)-oxidizing activity may be sustained in regions of the sediments that are depleted in O_2_.

## Introduction

In the Appalachian coal mining regions of the northeastern United States, acid mine drainage (AMD) arises when coal seam-associated iron sulfide phases are exposed to O_2_-rich fluids during or upon completion of mining activities (Baker and Banfield, 2003). Biogeochemical reactions in the subsurface between O_2_ and FeS phases result in O_2_-depleted, acidic fluids (typically pH 2.5–4.0 in the Appalachian coal mining regions) that contain high concentrations of sulfate, Fe(II), and other metals (Cravotta, 2008). Several systems have been identified in the Appalachian coal mining regions in which AMD emerges at the terrestrial surface and flows as a sheet with a depth of 0.5–1.0 cm (Senko et al., 2008; DeSa et al., 2010; Brown et al., 2011; Senko et al., 2011; Gouin et al., 2013). This flow regime enhances aeration of the fluids, and consequently the activities of acidophilic, aerobic Fe(II) oxidizing bacteria (FeOB). While abiotic Fe(II) oxidation is kinetically limited at the pH encountered in Appalachian coal mine-derived AMD-impacted systems, the solubility of Fe(III) is low, such that hydrolysis and precipitation of Fe(III) (hydr)oxides occurs (Regenspurg et al., 2004; Senko et al., 2008; DeSa et al., 2010; Brown et al., 2011; Gouin et al., 2013). With sustained emergence of AMD, FeOB activities give rise to massive deposits (tens to hundreds of cm deep) that are composed almost exclusively of biogenic Fe(III) (hydr)oxides and are referred to as “iron mounds” (Senko et al., 2008; Brown et al., 2011; Gouin et al., 2013). At an iron mound located in eastern Ohio (referred to as “The Mushroom Farm” or MF), we have observed the oxidative precipitation of approximately 10 mM Fe over a distance of 30 m after AMD emergence at the terrestrial surface (Gouin et al., 2013). At the MF system and elsewhere, iron mounds develop with no human intervention (Senko et al., 2008; DeSa et al., 2010; Brown et al., 2011; Gouin et al., 2013), so it has been proposed that engineered systems mimicking the sheet flow characteristics of iron mounds could be exploited for the inexpensive and sustainable removal of dissolved Fe(II) from AMD (Senko et al., 2008; DeSa et al., 2010; Brown et al., 2011), which remains the greatest threat to surface water quality in Appalachia (US EPA, 2006).

The Fe(III) hydroxides in iron mounds may accumulate quite rapidly. For instance, at the MF system, AMD began flowing over formerly pristine soil approximately 20 years ago (Cheryl Socotch, personal communication), and since then, a 8–10 cm thick Fe(III) (hydr)oxide crust has developed, indicating an iron mound “growth rate” of 0.4–0.5 cm/year. This rapid accumulation of Fe(III) (hydr)oxides poses a challenge to microorganisms mediating the oxidative precipitation of Fe. An implication of the rapid accumulation of Fe(III) (hydr)oxide precipitates is that the microorganisms mediating oxidative precipitation of Fe may become buried in Fe(III) phases that they produce. Similarly, O_2_ is likely to be depleted at the constantly rising sediment-water interface, giving rise to anoxic zones within the sediments. Anaerobic metabolism within iron mound sediments is likely to be predominantly Fe(III) or sulfate respiration (Burton et al., 2007; Sánchez-Andrea et al., 2011). These activities may be considered detrimental to the overall goal of oxidative precipitation of Fe, since these activities could result in the reductive re-release of Fe(II), or concentration of FeS phases near the terrestrial surface, which, if subsequently oxidized upon intrusion of O_2_, would result in release of more concentrated AMD (Johnson and Hallberg, 2002). However, the depth-dependent geochemical gradients and associated distributions of microorganisms in iron mounds and physicochemically similar systems remain unclear. As such, we evaluated the chemistry and microbiology at ≤1 cm depth intervals within the MF iron mound.

## Materials and methods

### Site description and field sampling

The MF AMD-impacted system is located in North Lima, Mahoning County, OH (40°56′09″N, 80°40′04″W). AMD from an abandoned subsurface mine has filled the basement of a now-vacant house, from which it emerges with pH 4.2 and 12 mM Fe(II) (Gouin et al., 2013). AMD that emerges from the basement window well of the vacant house flows as a sheet over the terrestrial surface, which has given rise to an iron mound covering an area of approximately 45 m^2^ (Gouin et al., 2013) that is composed of 7 mmol Fe/g (75% Fe(III) phases by mass, if Fe(OH)_3_ is assumed; Bertel and Senko, unpublished). All measurements and sample collection were conducted in a 0.5 m^2^ portion of the iron mound located approximately 6 m from AMD emergence. AMD continuously flows throughout the year as a sheet over this portion of the iron mound. A series of cores were collected from this region using 60 cc syringes with the leur ends removed. Upon recovery of the cores, they were covered with plastic wrap, sealed with vinyl tape, and placed on ice for transport to the laboratory, where they were extruded at approximately 1 cm intervals for subsequent mineralogical analysis by X-ray diffraction (XRD) and quantification of solid-associated Fe(II), total organic carbon (TOC), and total nitrogen (described below). Passive porewater samplers similar to those described by Spaulding and Brooks (2005) were prepared by melting agarose (5%) in deionized water with 1 mM KBr, which served as a tracer to determine complete equilibration of passive sampler fluid with the surrounding iron mound porewater. To cast the samplers, molten agarose was solidified within 60 cc syringes with the leur ends removed. The resulting agarose cylinders (referred to as “plugs”) with internal dimensions of the 60 cc syringes were subsequently inserted into holes left behind by coring. Two passive sampling plugs were deployed. Passive sampling “plugs” were incubated in the iron mound for one month before recovery, at which point, they had swelled to fill the entire void left behind by coring. Passive sampler “plugs” were transported to the laboratory and sectioned at 0.5 cm intervals before quantification of dissolved Fe(II) and sulfate (described below).

Cores used for microbial enumerations were collected in the same fashion, except that syringes were autoclaved and cores were covered with sterile aluminum foil for transport to the laboratory on ice. Core for nucleic acid-based microbial community analysis was collected using a sterile polypropylene core liner with a diameter of 2.5 cm. Cores intended for microbial enumerations were stored at 4°C for no more than 1 week before enumerations were initiated (described below). The core intended nucleic acid-based microbial community analysis was stored at −80°C before further processing (described below).

### Analytical techniques

After sectioning, passive sampling “plugs,” 0.5 cm sections were divided into quadrants. One quadrant was ground with a spatula and suspended in purified water for extraction of plug-associated sulfate and bromide, while a second quadrant was treated identically, except that it was suspended in 0.5 M HCl for extraction of plug-associated Fe(II). Plug-fluid suspensions were incubated overnight at room temperature, solids were removed from fluid by centrifugation, and aqueous sulfate (and bromide) and Fe(II) in the supernatant were quantified by ion chromatography (Dionex DX-120 system fitted with an IonPac AS14 column and conductivity detector; Dionex, Sunnyvale, CA) and ferrozine assay (Stookey, 1970), respectively. Iron mound porewater sulfate and iron concentrations were calculated based on water content of a given volume of agarose plug. We were unable to detect bromide in the passive sampler plugs, indicating complete equilibration of the plug-associated fluid with surrounding iron mound porewater. Solid associated Fe(II) in iron mound sediments was quantified by extracting Fe(II) from iron mound material using 0.5 M HCl (Lovley and Phillips, 1987), followed by centrifugation to remove solids, and Fe(II) in the supernatant was quantified by ferrozine assay (Stookey, 1970). Total organic C and total N associated with iron mound sediments were quantified using a PerkinElmer 2400 Series II CHNS/O Analyzer (PerkinElmer Inc.; Waltham, MA). The mineralogy of Fe(III) phases in the iron mound was determined by X-ray powder diffractometry (XRD) using a Phillips 3100 automated diffractometer using CuKα radiation, scanning at 2Θ of 2–70°, and accelerating voltage of 40 kV at 35 mA. Measurements of x-ray intensities were determined with 0.02° step size and 1 s counting time/step. Microprofiling of pH and DO were conducted using a Unisense (Unisense A/S, Aarhus, Denmark) microsensor system fitted with OX-N and PH-N DO and pH electrodes, respectively. Microprofiling of pH was conducted in the field, but since a DO electrode malfunctioned in the field, a core was obtained, returned to the laboratory, and DO microprofiling was subsequently conducted approximately 1.5 h after core collection.

### Microbial enumerations

To enumerate FeOB, a subsample of extruded core material (approximately 0.3 g) was suspended in a solution containing 14 mM (NH_4_) _2_SO_4_ and 2 mM MgSO_4_ (pH adjusted to 3.5 with H_2_SO_4_), serially diluted in the same solution, and spread on solid medium-containing plates. The medium used for FeOB enumerations was based on that described by Johnson (1995), and contained 25 mM FeSO_4_, 14 mM (NH_4_)_2_SO_4_, 2 mM MgSO_4_, 0.25 g/l trypticase soy broth, vitamins and trace metals (Tanner, 1997). The pH of the medium was adjusted to 3.5 with H_2_SO_4_, and solidified with agarose (20 g/l). Plates were incubated at room temperature in darkness, and FeOB colony forming units (CFU) were counted based on the formation of rust-colored colonies.

To enumerate Fe(III) reducing bacteria (FeRB), a subsample of the extruded core material (approximately 0.3 g) was transferred to an anoxic glovebag (Coy Laboratory Products, Grass Lake, MI). Sediment was suspended in an acidophilic, anaerobic FeRB medium described by Senko et al. (2009) that contained 25 mM Fe_2_(SO_4_)_3_, 10 mM (NH_4_)_2_SO_4_, 2 mM MgSO_4_, 5 mM glucose, 0.5 g/l trypticase soy broth, vitamins and trace metals (Tanner, 1997). The pH of the medium was adjusted to 4.2 with NaOH, which caused the formation of Fe(III) (hydr)oxide precipitate in the medium. After preparation of the initial suspension of iron mound sediment in the FeRB medium, the suspension was serially diluted in a three-tube most probable number (MPN) series (Colwell, 1979) and incubated at room temperature in the dark. MPN series were scored based on the accumulation of ≥4 mM Fe(II) in the medium.

To enumerate total bacterial abundances present in the MF iron mound sediments, Fe(III) (hydr)oxides were first removed by washing with 0.3 M ammonium oxalate (pH adjusted to 3.0 with oxalic acid; Nicomrat et al., 2006; Senko et al., 2008). After ammonium oxalate washing, cell pellets were resuspended in phosphate-buffered saline solution, immobilized on a Isopore membrane filter (0.4 μm pore size; Millipore, Billerica, MA), attached to microscope slides, and stained with 4′,6-diamidino-2′-phenylindole (DAPI). Cells were visualized using an Olympus BX53 fluorescent microscope (Olympus America, Inc.; Center Valley, PA). Cell abundances were determined based on the average of 100 fields of view. To evaluate the possibility that the ammonium oxalate-washing step could damage cells and confound DAPI-based enumerations, cells from a late log phase culture of *Shewanella oneidensis* MR-1 were subjected to the ammonium oxalate washing procedure described above. Similar abundances of cells were observed in the DAPI-stained, ammonium oxalate cell suspension as in a DAPI-stained, unwashed cell suspension, indicating that the ammonium oxalate-washing step did not interfere with visualization of DAPI-stained cells.

### Nucleic acid-based microbial community characterization

In preparation for nucleic acid-based microbial community analysis, cores were removed from the −80°C freezer, and a small rotating saw was used to cut the core barrel length-wise. The halves of the core barrel were separated, and since the frozen sediments adhered to the core barrel, the interior of the sediment core was exposed. Samples were collected at 1 cm intervals from the interior of the core using sterile spatulas, and Fe(III) from the iron mound sediments was removed using 0.3 M ammonium oxalate as described above. Genomic DNA was extracted from the remaining material using MoBio (MoBio Laboratories, Inc., Carlesbad, CA) PowerBiofilm DNA isolation kits according to the manufacturer's instructions. Partial 16S rRNA gene sequences were obtained using tag-encoded FLX amplicon pyrosequences at Molecular Research LP (Shallowater, TX). The 16S universal primers based on *Escherichia coli* 16S rRNA gene positions 515 and 806 were used for a single-step 30 cycle PCR using HotStarTaq Plus Master Mix Kit (Qiagen, Valencia, CA) under the following conditions: 94°C for 3 min, followed by 28 cycles of 94°C for 30 s, and 53°C for 40 s and 72°C for 1 min, followed by a final 5 min elongation step at 72°C. All PCR amplicon products were mixed in equal concentrations and purified using Agencourt Ampure beads (Agencourt Bioscience Corporation, MA, USA). Samples were sequenced following the manufacturer's instructions using Roche (Roche Diagnostics Corp., Indianapolis, IN) 454 FLX titanium instruments and reagents. Upon obtaining sequence data, barcodes and primers were removed from sequences, and chimeras, sequences of <200 bp, sequences with ambiguous base calls, and/or sequences with homopolymer runs of >6 bp were removed from libraries (Gontcharova et al., 2010). Nucleotide sequence libraries from each depth have been submitted to the Sequence Read Archive (SRA) under run accession numbers SRR1206276 (0–1 cm depth interval), SRR1206277 (1–2 cm depth interval), SRR1206278 (2–3 cm depth interval), SRR1206279 (3–4 cm depth interval), SRR1206280 (4–5 cm depth interval), SRR1206281 (5–6 cm depth interval), SRR1206282 (6–7 cm depth interval), SRR1206283 (7–8 cm depth interval), SRR1206284 (8–9 cm depth interval), SRR1206285 (9–10 cm depth interval).

Standard rarefaction curves (based on 97% sequence similarity), Shannon, and Chao1 diversity indices were developed for sequence libraries from each depth interval using the Ribosomal Database Project-II (RDP-II) Pyrosequencing Pipeline (Cole et al., 2009). Further sequence processing was performed using the QIIME software package (Caporaso et al., 2011) in the MacQIIME environment (http://www.wernerlab.org/software/macqiime) using default parameters. Operational taxonomic units (OTU_0.03_) were determined at 97% sequence identity and picked using QIIME scripts (Edgar, 2010). Taxonomic assignments were subsequently made to OTU_0.03_ using the RDP-II classifier function while still in the QIIME environment (Wang et al., 2007). OTU_0.03_ comprising ≥0.5% of sequences in libraries from each depth interval were identified and compared to sequences contained in the National Center for Biotechnology Information (NCBI) database using the Basic Local Alignment Search Tool (BLASTn; Altschul et al., 1997). The PyNAST algorithm was used to align sequences against the Greegenes core sequence set (DeSantis et al., 2006), and a phylogenetic tree containing OTU_0.03_ from all depth intervals was constructed in QIIME. The OTU_0.03_ table from each sample was interatively rarified using jack-knife sampling to 7844 sequences, and distance matrices were developed using the weighted and unweighted UniFrac metrics (Lozupone et al., 2007). Clustering of microbial communities associated with different depth intervals was evaluated by construction of unweighted pair group method with arithmetic mean (UPGMA) trees based on distance matrices produced using UniFrac (Lozupone et al., 2007).

## Results and discussion

### Geochemical and mineralogical gradients in MF iron mound sediments

**Figure 1:**
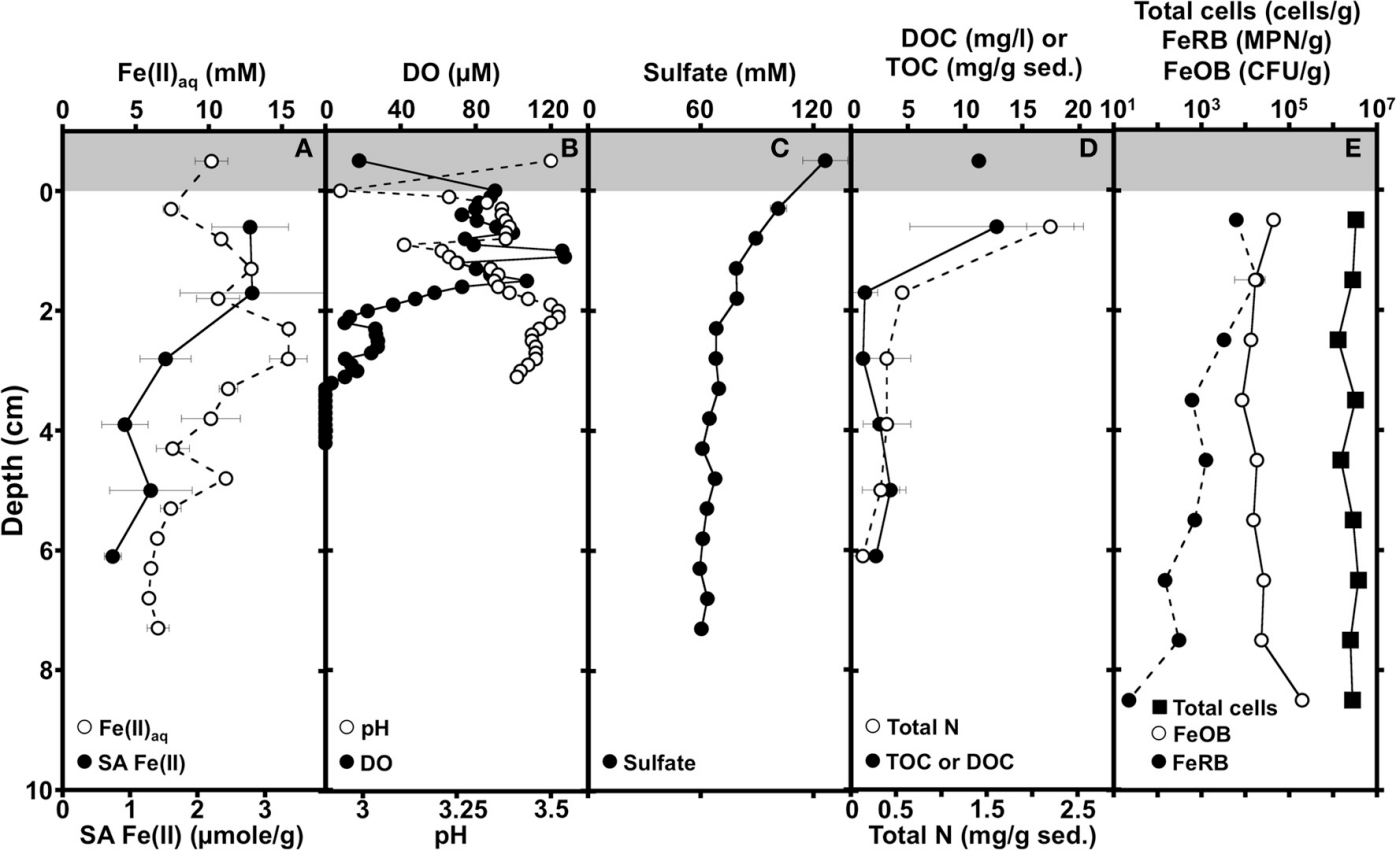
**Depth-dependent profiles of dissolved (Fe(II)_aq_) and solid-associated Fe(II) (0.5 M HCl-extractable; SA Fe(II)) (A), dissolved oxygen (DO) and pH (B), sulfate (C), Dissolved organic carbon (DOC) of overlying AMD, total organic carbon (TOC), and total nitrogen of sediments (D), and abundances of total cells, culturable Fe(II) oxidizing bacteria (FeOB), and culturable Fe(III) reducing bacteria (FeRB) in iron mound sediments (E)**. Shading at the top of the panels illustrates AMD overlying the sediments. Error bars represent one standard deviation.

The dissolved Fe(II) concentration at the 0–0.5 cm depth interval was lower than that of the overlying fluid and pH was lowest at the sediment-water interface, indicating oxidative precipitation of Fe(II) (Equation 1) in this region of the iron mound sediments (Figures 1A,B).

(1) $$4{\text{Fe}}^{2+}+{\text{O}}_{2}+10{\text{H}}_{2}\text{O}\to 4\text{Fe}{\left(\text{OH}\right)}_{3}+8{\text{H}}^{+}$$

Dissolved Fe(II) increased with depth concomitantly with depletion of O_2_, reaching a maximum Fe(II) concentration of 15 mM approximately 2.5 cm below the sediment water interface (Figures 1A,B). The maximum aqueous Fe(II) concentration within the iron mound sediments exceeds that of the overlying AMD (Figure 1A), indicating that reductive dissolution of iron mound Fe(III) phases is occurring in this depth interval. With increasing depth below approximately 2.5 cm, the Fe(II) concentration decreased (Figure 1A). Fe(II) adsorbs strongly to Fe(III) (hydr)oxide phases (e.g., Jeon et al., 2003; Jang et al., 2008), so the MF iron mound sediments themselves represent a potential pool of Fe(II). Indeed, we detected a large fraction of solid associated (0.5 M HCl-extractable) Fe(II) adsorbed to sediments in the upper 2 cm of the iron mound (Figure 1A). However, the solid associated Fe(II) content of sediments diminished with depth (Figure 1A), indicating that decrease in aqueous Fe(II) concentration below 2.5 cm may not be exclusively due to adsorption of Fe(II) on Fe(III) (hydr)oxide phases in the iron mound sediment. Porewater sulfate concentration was lower than the overlying AMD, and decreased slightly in the depth interval between the sediment-water interface and approximately 2 cm, but changed little below this depth (Figure 1C). Since sulfate depletion occurred in a depth interval that contained abundant dissolved oxygen (Figure 1C), it is unlikely attributable to sulfate reduction. Rather, the removal of sulfate from solution could be attributable to adsorption of sulfate on freshly precipitated Fe(III) phases (Parfitt and Smart, 1977) or incorporation into Fe(III) (hydr)oxysulfates such as schwertmannite (Bigham et al., 1990; Burton et al., 2007), concurrently with Fe(II) oxidation near the sediment-water interface. TOC and total N were highest in the shallowest portion of the iron mound (Figure 1D), suggesting primary productivity in this region of the iron mound. With increasing depth within the iron mound, both TOC and total N were depleted (Figure 1B).

**Figure 2:**
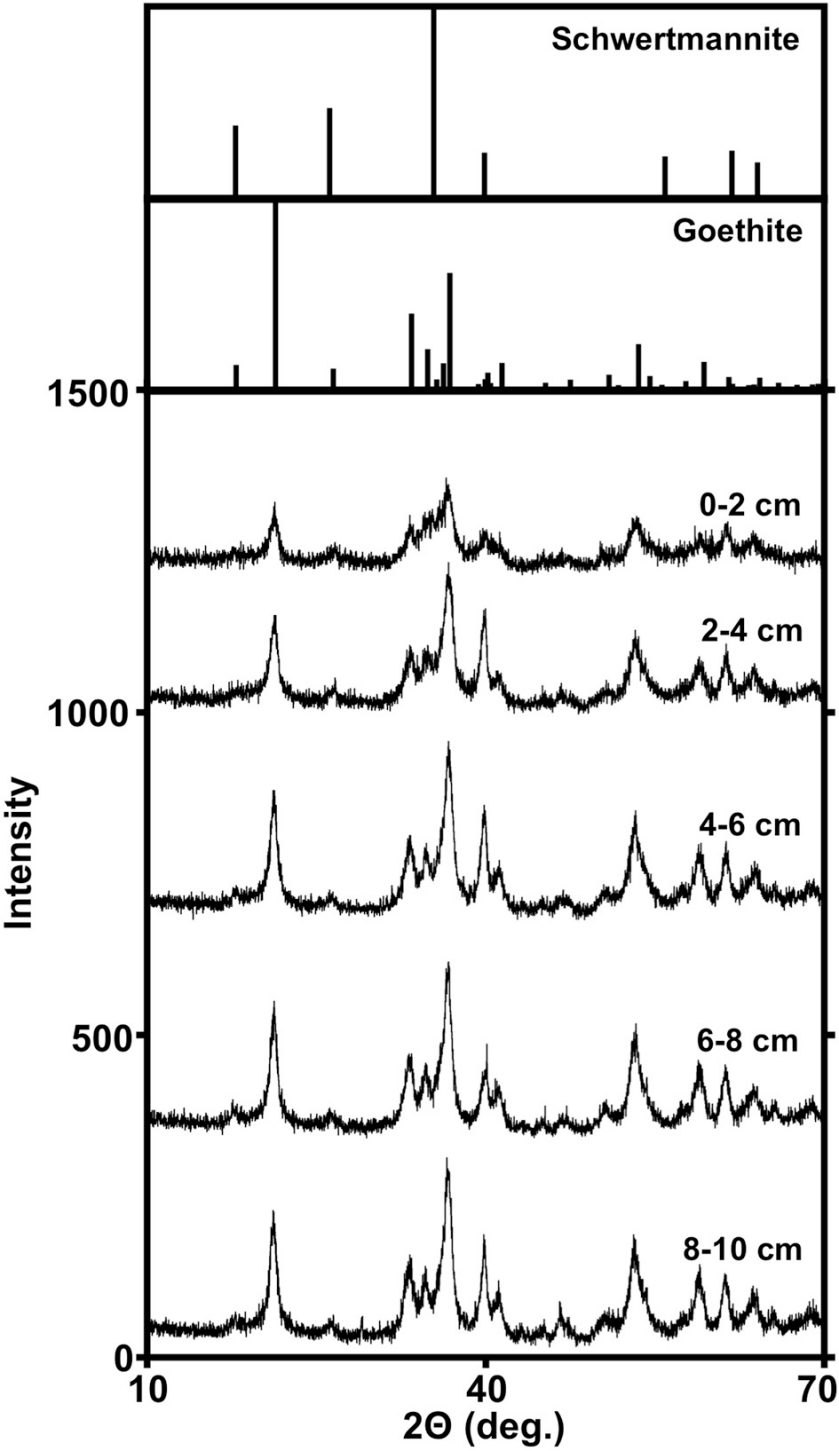
**Powder X-ray diffraction patterns of Fe(III)-rich phases recovered from different depths within the MF iron mound**. Reference diffraction patterns of schwertmannite and goethite are from The American Mineralogist Crystal Structure Database (Downs and Hall-Wallace, 2003).

Geochemical evaluations of the MF iron mound indicated that aerobic microbial activities were occurring most extensively in the upper 2.5 cm of the sediments, but Fe(III) reduction was also occurring in this region, despite the presence of dissolved O_2_ to a depth of approximately 3 cm (Figures 1A,B). XRD patterns obtained from sediments in the 0–2 cm depth interval contained broad poorly resolved peaks indicative of goethite (α-FeOOH) of small grain size or poorly-crystalline Fe(III) phases, such as hydrous ferric oxide or schwertmannite (Fe_8_O_8_(OH)_6_(SO_4_)•*n*H_2_O), which is frequently observed as the predominant Fe(III) phase formed by biological or abiotic oxidation of Fe(II) in AMD with pH from 2.8 to 4.5 (Figure 2; Bigham et al., 1996a,b; Burgos et al., 2012). With increasing depth in the iron mound, we observed higher peak to background ratios and XRD patterns indicating goethite (Figure 2). This depth-dependent development of goethite from poorly crystalline phases is typical of AMD-derived Fe(III) (hydr)oxide sediments and non-acidic systems, and may be induced by Fe(III)- and/or sulfate-reducing bacterial activities (Hansel et al., 2003; Burton et al., 2007; Bertel et al., 2012). While no iron sulfide phases were detected by XRD, biogenic sulfide reacts rapidly with Fe(III) phases in such systems (Neal et al., 2001; Poulton et al., 2004). As such, any sulfide produced by sulfate reducing bacterial activities would be unlikely to accumulate, but would be oxidized by Fe(III) to a variety of partially oxidized S species (Neal et al., 2001; Poulton et al., 2004).

### Culture-dependent characterization of microbial communities associated with MF iron mound sediments

Both geochemical and mineralogical evaluations of the MF iron mound suggested concomitant aerobic and anaerobic activities in the upper ~3 cm of the iron mound sediments, though indications of anaerobic activity [i.e., Fe(II) accumulation or sulfate depletion] below this depth could not be detected. As such, we sought to evaluate the microbial communities associated with the iron mound at discrete depth intervals. Total abundances of microbial cells were uniform throughout the column (Figure 1E). Similarly, culturable aerobic Fe(II) oxidizing bacterial (FeOB) abundances were similar throughout the iron mound sediments, though they were most abundant in the 0–1 cm interval and 9–10 cm interval (Figure 1E). Culturable Fe(III) reducing bacterial (FeRB) abundances increased slightly from the 0–1 cm depth interval to the 1–2 cm depth interval [where dissolved Fe(II) concentrations indicated Fe(III) reduction was occurring], and subsequently decreased in abundance with increasing depth in the iron mound (Figure 1E). The relatively uniform abundances of aerobic FeOB and depth-dependent decrease in FeRB abundances was somewhat surprising, since dissolved O_2_ was depleted below approximately 3 cm in the iron mound sediments. While the abundance of culturable FeOB has been illustrated to be an indicator of rates of Fe(II) oxidation in iron mound sediments (Senko et al., 2011), the uniform distribution of FeOB and diminished abundance of FeRB with depth in the MF iron mound sediments may be a reflection of the metabolic versatility of Fe metabolizing acidophilic microorganisms. For instance, several organisms classified as aerobic FeOB may also respire Fe(III) or partially reduced S species under anoxic conditions (Johnson and McGinness, 1991; Pronk et al., 1992; Küsel et al., 1999; Hedrich et al., 2011), so the aerobic FeOB that were detected may have been metabolizing anaerobically in O_2_-depleted regions of the iron mound. Similarly, no obligately anaerobic acidophilic FeRB have been recovered in culture. While acidophilic/acid-tolerant SRB capable of Fe(III) reduction have been isolated from AMD-impacted systems, it remains unclear whether these organisms can couple Fe(III) reduction to growth (Senko et al., 2009; Alazard et al., 2010). Several aerobic organotrophic acidophilic microorganisms capable of Fe(III) respiration have been isolated (Johnson and McGinness, 1991; Pronk et al., 1992; Küsel et al., 1999; Hedrich et al., 2011). As such, the greater abundance of culturable FeRB that we detected in shallower regions of the MF iron mound may be a reflection of the increased availability of organic carbon in these portions of the sediment (Figure 1D), since we enumerated FeRB using glucose as an electron donor. Alternatively, the higher numbers of FeRB in the shallower sediments may be attributable to the abundance of poorly crystalline Fe(III) phases, which may be more susceptible to bioreduction than goethite (Burton et al., 2007). The recovered FeOB and FeRB comprised ≤1% of total cells detected by microscopic counting of DAPI-stained cells, and similarly poor recoveries of cells in culture have been reported from other AMD-impacted systems (Hallberg et al., 2006). Despite the relatively poor recovery of Fe metabolizing organisms, it remains notable that FeOB and FeRB were in close spatial association with each other within the iron mound sediments.

### Nucleic acid-based characterization of microbial communities associated with MF iron mound sediments

**Figure 3:**
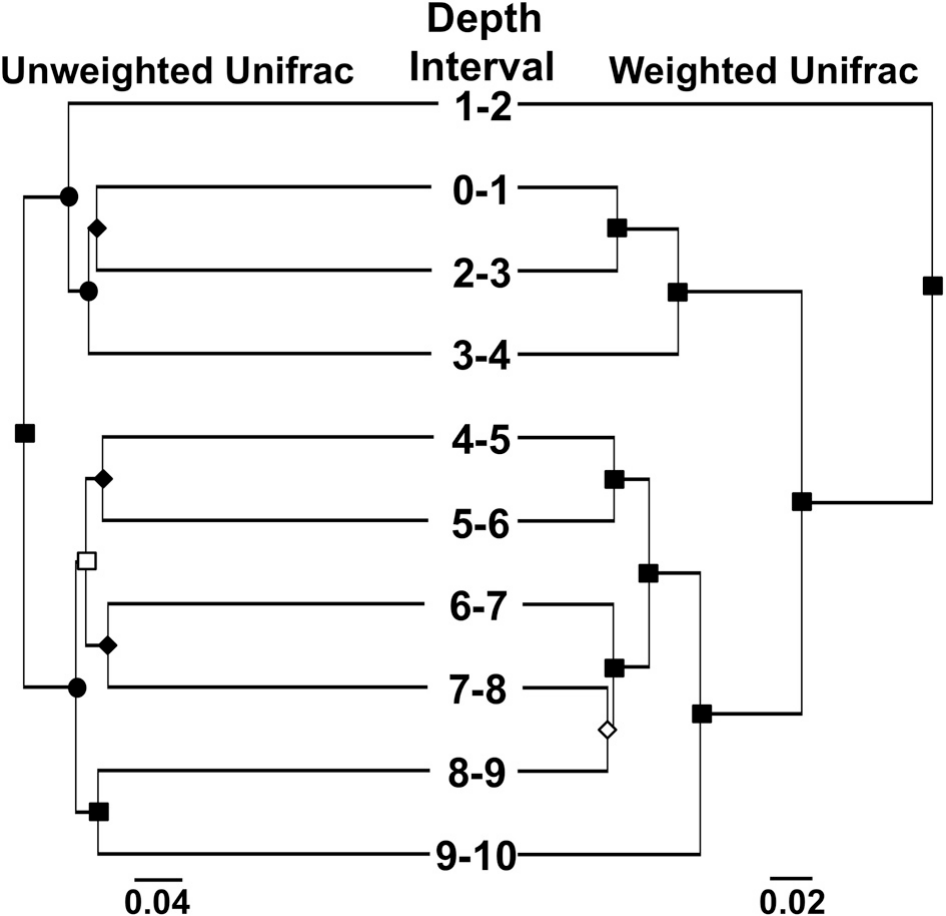
**UPGMA trees showing clustering of microbial communities associated with different depth intervals in the MF iron mound using the weighted and unweighted Unifrac metrics**. Symbols on nodes: ■, •, ♦, □, and ◊ represent ≥99%, 90–99%, 80–89%, 70–79%, and 50–59% jackknife support.

We used “next generation” DNA sequencing to evaluate partial (average read length 264 bp) 16S rRNA gene sequences from microorganisms associated with MF iron mound sediments at 1 cm depth intervals. The number of sequences recovered from each depth interval ranged from 7844 to 18,385 (Table 1). Non-parametric indicators of community diversity (Chao1 and Shannon indices) revealed that the diversity of the communities was uniformly low throughout the sediments (Table 1), in comparison to nearby AMD-unimpacted soil (Brantner and Senko, unpublished). UPGMA clustering of microbial communities using the Unifrac metric for comparison of community composition (Lozupone et al., 2007) revealed depth-dependent segregation of microbial communities within the MF iron mound sediments (Figure 3). Microbial communities at depths below 4 cm clustered together regardless of whether the unweighted or weighted Unifrac metrics were used to compare the communities (Figure 3). While the microbial community from the 1–2 cm depth interval did not cluster with those associated with depths below 4 cm, it also did not cluster with communities associated with the 0–1 and 2–4 cm depth intervals (Figure 3), likely due to the high relative abundance of chloroplast-attributable 16S rRNA gene sequences detected at this depth interval (discussed in more detail below). Clustering of microbial communities based on position above and below 4 cm depth indicated that the availability of O_2_ exerted some control on the composition of the microbial communities.

**Table 1:** **Information on 16S rRNA gene sequence libraries and non-parametric diversity estimates of microbial communities from MF iron mound**.

**Depth interval**	**Number of sequences**	**Number of OTU_0.03_**	**Chao1 index**	**Shannon index**
0–1 cm	18,385	668	994	4.44
1–2 cm	14,350	457	737	4.02
2–3 cm	10,718	522	824	4.35
3–4 cm	8654	523	653	4.09
4–5 cm	8529	423	491	3.99
5–6 cm	15,694	673	968	4.49
6–7 cm	13,553	652	866	4.53
7–8 cm	8204	498	637	3.98
8–9 cm	7844	366	418	3.91
9–10 cm	17,645	637	936	4.33

**Figure 4:**
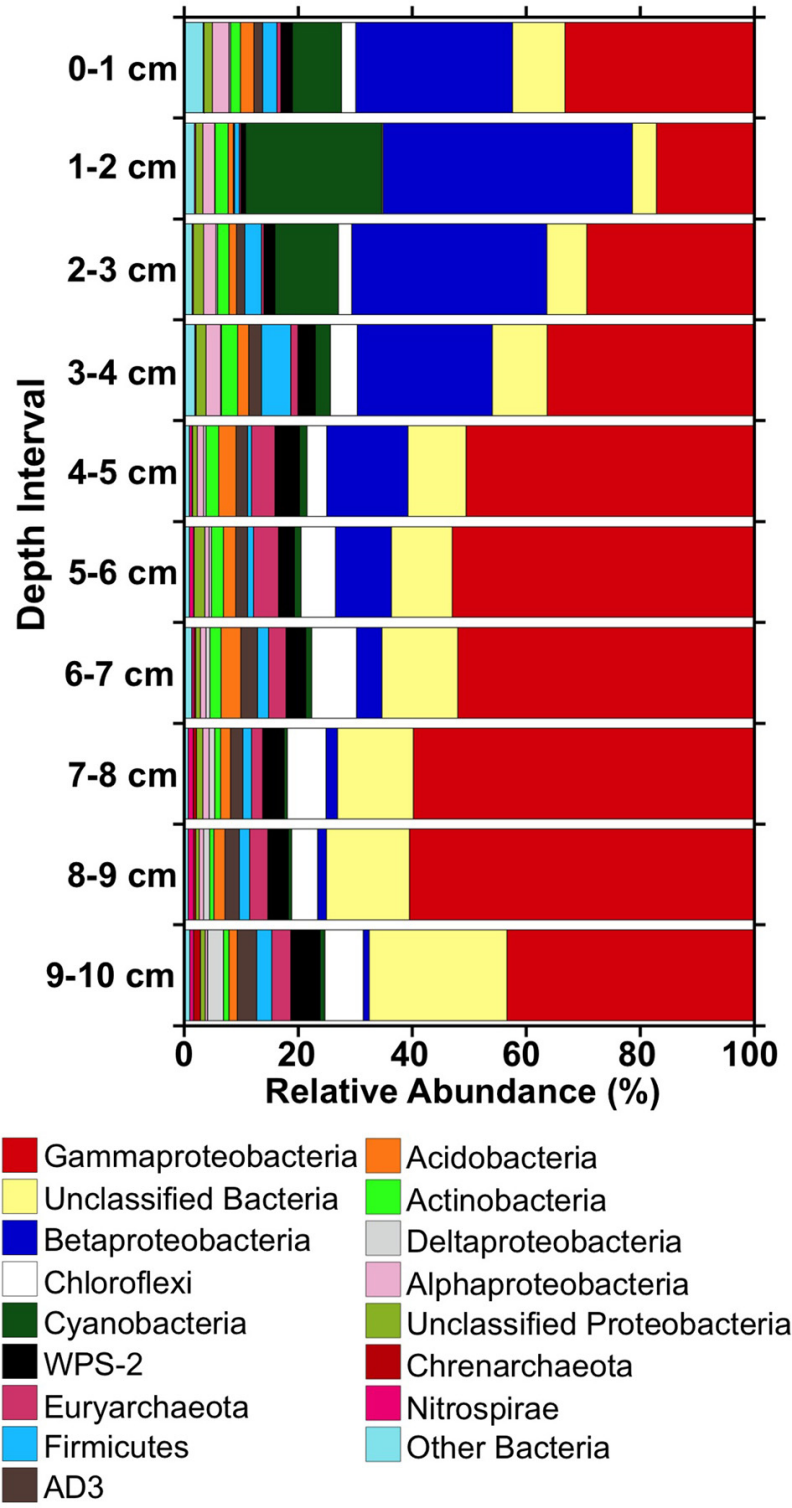
**Relative abundances of 16S rRNA gene sequences detected in libraries from different depths within the MF iron mound at phylum-level and class-level (in the cases of the Proteobacteria) taxonomic resolution**.

When viewed from the phylum level, Gammaproteobacterial phylotypes were abundant throughout the iron mound sediments, comprising approximately 20% or more of the sequences in libraries obtained from each depth interval, and comprised a slightly higher fraction of the microbial community at depths below 4 cm (Figure 4). Betaproteobacteria- and Cyanobacteria-affiliated phylotypes were prominent representatives of sequence libraries recovered from the upper 4 cm of the iron mound, but decreased in relative abundance below 4 cm (Figure 4). Unclassifiable Bacteria- and, to a lesser extent, Chloroflexi- and Euryarchaeota-affiliated phylotypes increased in relative abundance with depth in the iron mound sediments (Figure 4).

**Figure 5:**
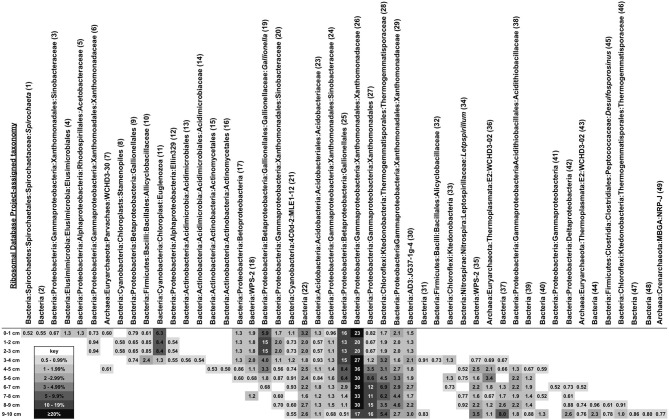
**Heatmap showing relative abundances and Ribosomal Database Project-derived taxonomic identities of OTU_0.03_ comprising ≥0.5% of total OTU_0.03_ in the MF iron mound at different depth intervals**. Numbers in parentheses after taxonomic assignments refer to results (shown in Table 2) of BLASTn searches of each OTU_0.03_ represented in the figure.

Phylotypes attributable to WPS-2, Firmicutes, AD3, Acidobacteria, and Actinobacteria each comprised 1–5% of total sequences in the iron mound sediments, but clear depth-dependent trends in their relative abundances were not observed (Figure 4). Anywhere from 19 to 26 OTU_0.03_ represented ≥0.5% of sequences in libraries derived from each depth interval, and comprised 72–78% of the total sequences in each library (Figure 5). Of these OTU_0.03_, a set of nine OTU_0.03_ attributable to Acidobacteria, Gammaproteobacteria, Betaproteobacteria, Chloroflexi, AD3, and unclassifiable Bacteria were detected throughout the iron mound sediments at abundances ≥0.5% of respective sequence libraries (Figure 5). To make more specific taxonomic assignments to these numerically prominent phylotypes, we compared OTU_0.03_ represented in Figure 5 to sequences contained in the GenBank database using BLASTn (Altschul et al., 1997; Table 2). The majority of these OTU_0.03_, particularly those detected in the upper portion of the iron mound, as well as those assignable to the most abundant phyla (i.e., Gamma- and Betaproteobacteria, Cyanobacteria, and unclassifiable Bacteria), were 96–100% similar to 16S rRNA gene sequences detected in AMD-impacted systems (Figure 5, Table 2).

**Table 2:** **Most closely related (based on BLASTn-determined similarity) 16S rRNA gene sequences from environmental surveys and cultured microorganisms to OTU_0.03_ comprising ≥0.5% of sequences in libraries recovered from different depth in the MF iron mound**.

**OTU #**	**Nearest environmental 16S rRNA gene sequence**	**Nearest cultured 16S rRNA gene sequence**
1	AMD-impacted Río Tinto (99%; FN867143; Amaral-Zettler et al., 2011)	*Spirochaeta aurantia* from freshwater (94%; AJ565432; Hahn et al., 2004)
2	AMD-impacted Río Tinto (98%; FN863828; Amaral-Zettler et al., 2011)	None found
3	“Iron snow” in acidic mine lake (99%; HE604017; Lu et al., 2013)	*Steroidobacter denitrificans* FS, NO_3_^−^ reducer from anoxic sludge (94%; NR_044309) (Fahrbach et al., 2008)
4	Abandoned Cu mine (99%; JQ217995; Falteisek and Cepička, 2012)	*Halothiobacillus kellyi* BII-1, thermophilic S oxidizer (84%; NR_025030; Sievert et al., 2000)
5	“Iron snow” in acidic mine lake (99%; HE604030; Lu et al., 2013)	Acidophilic FeOB C4H7 (99%; JX869450; Wu et al., 2013)
6	Abandoned Cu mine (99%; JQ217802; Falteisek and Cepička, 2012)	*Metallibacterium* X11 adidophilic S_2_O_3_^2-^ oxidizer (95%; HE858262; Delavat et al., 2012)
7	AMD-impacted Río Tinto (96%; FN866063; Amaral-Zettler et al., 2011)	None found
8	Arctic stream epilithon (97% FJ849138; Larouche et al., 2012)	*Elphidium aculeatum* A75.46 chloroplast (85%; HM213365; Pillet et al., 2011)
9	Abandoned Cu mine (99%; JQ217544; Falteisek and Cepička, 2012); AMD iron mound (99%; HQ420151; Brown et al., 2011)	*Ferrovum myxofaciens* EHS8, acidophilic FeOB (97%; KC155322; Hedrich et al., 2009); *Ferrovum myxofaciens* PSTR, acidophilic FeOB (97%; EF133508; Hallberg et al., 2006)
10	AMD-impacted sediment (99%; EF409850; Yin et al., 2008)	Acidophilic FeOB/FeRB iFeo-D4-31-CH (94%; FN870336; Lu et al., 2010)
11	AMD-impacted Río Tinto (99%; FN862195; Amaral-Zettler et al., 2011)	*Euglena mutabilis* SAG 1224-9b chloroplast (96%; AY626044; Milanowski et al., 2006)
12	Metal sulfide mine AMD (99%; GU979565; Hao et al., 2010)	Rhizobiales strain A48, neutrophilic FeRB (94%; AB081581; Satoh et al., 2002)
13	AMD-impacted Río Tinto sediment (99%; JF737887; García-Moyano et al., 2012)	*Aciditerrimonas ferrireducens*, thermoacidophilic FeRB (92%; AB517669; Itoh et al., 2011)
14	Acidic mine lake (99%; KC619609; Santofimia et al., 2013)	Acidophilic heterotrophic FeOB Py-F3 (96%; KC208497; Kay et al., 2013)
15	Acidic wetland soil (99%; GQ203360; Kopecky et al., 2011)	*Actinoallomurus* sp. 645152, acidophilic organotroph (96%; AB604840; Murmatsu et al., 2011)
16	Acidic mine lake sediments (99%; FN870199; Lu et al., 2010)	Acidophilic FeOB A4F6 (93%; JX869415; Wu et al., 2013)
17	AMD iron mound (97%; HQ420151; Brown et al., 2011); AMD-impacted Río Tinto (97%; FN867145; Amaral-Zettler et al., 2011)	*Ferrovum myxofaciens* EHS8, acidophilic FeOB (97%; KC155322; Hedrich et al., 2009); *Ferrovum myxofaciens* PSTR, acidophilic FeOB (97%; EF133508; Hallberg et al., 2006)
18	“Iron snow” in acidic mine lake (99%; HE604029; Lu et al., 2013)	*Arhodomonas* sp. Seminole, aerobic halophile (84%; JX099567; Dalvi et al., 2012)
19	Abandoned Cu mine (99%; JQ217975; Falteisek and Cepička, 2012)	*Leptothrix ochracea* SCGC AAA018-M4 FeOB (98%; HQ290506; Fleming et al., 2011)
20	AMD-impacted Río Tinto (99%; FN860398; Amaral-Zettler et al., 2011)	Acidophilic FeOB A4F5 (98%; JX869415; Wu et al., 2013)
21	Acidic hot spring (97%; JF280561; Bohorquez et al., 2012)	Anaerobic bacterium BSV83 (86%; AJ229227; Hengstmann et al., 1999)
22	AMD-impacted Río Tinto (99%; FN866617; Amaral-Zettler et al., 2011)	*Lysobacter* sp. AP7 (82%; EU374884; Alonso-Gutiérrez et al., 2009)
23	Abandoned Cu mine (99%; JQ218102; Falteisek and Cepička, 2012)	Acidophilic, organotrophic Acidobacteriaceae CH1 from AMD (97%; DQ355184; Diaby et al., 2007)
24	AMD-impacted Río Tinto sediment (99%, HQ730615; Sánchez-Andrea et al., 2011)	Acidophilic FeOB A4F5 (98%; JX869414; Wu et al., 2013)
25	Abandoned Cu mine (99%; JQ217544.1; Falteisek and Cepička, 2012)	*Ferrovum myxofaciens* EHS8, acidophilic FeOB (99%; KC155322; Hedrich et al., 2009); *Ferrovum myxofaciens* PSTR, acidophilic FeOB (99%; EF133508; Hallberg et al., 2006)
26	AMD biofilm (99%; JX297618.1; Guo et al., 2013)	*Metallibacterium* X11 adidophilic S_2_O_3_^2−^ oxidizer (99%; HE858262; Delavat et al., 2012); *Metallibacterium scheffleri* DKE6, acidophilic facultative FeRB (98%; HQ909259.1; Ziegler et al., 2013)
27	AMD-impacted Río Tinto (99%; FN862147; Amaral-Zettler et al., 2011)	*Thiohalophilus thiocyanatoxydans* HRhD 2, halophilic S oxidizer (91%; NR_043875; Sorokin et al., 2006)
28	Volcanic deposits (88%; AY917857; Gomez-Alvarez et al., 2007)	*Thermogemmatispora onikobensis*, thermophilic organotroph (86%; AB547912; Yabe et al., 2011)
29	Abandoned Cu mine (100%; JQ217580; Falteisek and Cepička, 2012)	*Metallibacterium* X11 adidophilic S_2_O_3_^2−^ oxidizer (100%; HE858262; Delavat et al., 2012); Acidophilic Fe(II) oxidizer C4C8 (97%; JX869446; Wu et al., 2013); *Metallibacterium scheffleri* DKE6, acidophilic facultative FeRB (97%; HQ909259.1; Ziegler et al., 2013)
30	“Iron snow” in acidic mine lake (99%; HE604014; Lu et al., 2013)	Acidophilic FeOB A10G4 (99%; JX869422; Wu et al., 2013)
31	Reject coal-impacted soil (97%; AF523920; Brofft et al., 2002)	*Moorella* sp. 64_FGQ, thermophlilic FeRB (87%; GQ872425; Nepomnyashchaya et al., 2012)
32	Metal sulfide mine AMD (99%; GU979565; Hao et al., 2010)	*Alicyclobacillus acidoterrestris* C-ZJB-12-17, thermoacidophilic (96%; KC193190; Zhang et al., 2013)
33	Volcanic deposits (90%; AY425781; Gomez-Alvarez et al., 2007)	*Thermogemmatispora onikobensis*, thermophilic organotroph (85%; AB547912; Yabe et al., 2011)
34	AMD-impacted Río Tinto (99%; FN863733; Amaral-Zettler et al., 2011)	*Leptospirillum ferrooxidans* C2-3 acidophilic FeOB (99%; NR_074963) Fujimura et al., 2012
35	AMD-impacted Río Tinto sediment (100%; JF737887; García-Moyano et al., 2012)	Chloroflexi SCGC AAA007-G23, marine S oxidizer (82%; HQ675468; Swan et al., 2011)
36	AMD-impacted creek (99%; HE653802; Volant et al., 2012)	*Methanomassiliicoccus luminyensis* B10, methanogen (85%; HQ896499; Dridi et al., 2012)
37	Anoxic rice field soil (89%; FM956256; Gan et al., 2012)	*Thermoactinomyces vulgaris* 55N1-5, thermophilic organotroph (88%; JN366723; Lima et al., 2012)
38	AMD-impacted Río Tinto (99%; FN862217; Amaral-Zettler et al., 2011)	*Acidithiobacillus ferrivorans* SS3, FeOB/facultative FeRB (99%; NR_074660; Liljeqvist et al., 2011)
39	Abandoned Cu mine (99%; JQ218054; Falteisek and Cepička, 2012)	*Clostridium ghonii* 2447_6, fermentative anaerobe (76%; JN048963; Ryzinska-Paier et al., 2011)
40	Deep granitic fracture water (88%; 7150D1B75; Sahl et al., 2008)	*Pelotomaculum terephthalicicum* JT strain J, syntroph (85%; NR_040948; Qiu et al., 2004)
41	AMD biofilm (95%; JX297618.1; Guo et al., 2013)	*Metallibacterium* X11 adidophilic S_2_O_3_^2−^ oxidizer (94%; HE858262; Delavat et al., 2012); Acidophilic FeOB C4C8 (94%; JX869446; Wu et al., 2013); *Metallibacterium scheffleri* DKE6, acidophilic facultative FeRB (94%; HQ909259.1; Ziegler et al., 2013)
42	Alpine tundra soil (94%; FJ570063; Zinger et al., 2009)	*Myxobacterium* sp. KC, humic substance oxidizer (93%; AF482687; Coates et al., 2002)
43	AMD-impacted creek (99%; HE653802; Volant et al., 2012)	*Methanomassiliicoccus luminyensis* B10, methanogen (85%; HQ896499; Dridi et al., 2012)
44	Anoxic rice field soil (87%; FM956256; Gan et al., 2012)	*Thermoactinomyces vulgaris* 55N1-5, thermophilic organotroph (86%; JN366723; Lima et al., 2012)
45	Acidic mine lake (93%; KC619609; Santofimia et al., 2013)	*Desulfosporosinus* sp. GBSRB4.2 (99%; EU839714; Senko et al., 2009)
46	Arctic fell-field soil (94%; EF221592; Yergeau et al., 2007)	*Ktedobacter racemifer* SOSP1-21, aerobic organotroph (88%; AM180160; Cavaletti et al., 2006)
47	AMD-impacted Río tinto (97%; FN860399; Amaral-Zettler et al., 2011)	Chloroflexi SCGC AAA007-G23, marine S oxidizer (79%; HQ675468; Swan et al., 2011)
48	AMD-impacted Río Tinto (99%; FN860359; Amaral-Zettler et al., 2011)	*Lysobacter* sp. AP7 (80%; EU374884; Alonso-Gutiérrez et al., 2009)
49	Acidic bog (99%; JQ807564; Lin et al., 2012)	Crenarchaeote OS70 (87%; EU239962; De la Torre et al., 2008)

OTU numbers in the left hand column refer to OTU number designations in Figure 5. Percent similarities, GenBank accession numbers, and appropriate references of sequences are provided in parentheses.

The abundant Gammaproteobacterial OTU_0.03_ were related to phylotypes detected in AMD-impacted systems and were affiliated with the order Xanthomonadales, using the RDP-II classifier function (Figure 5, Table 2), which have been previously detected in iron mound systems (Senko et al., 2008). The most abundant phylotype detected in the MF iron mound, regardless of depth, was 98–99% similar to acidophilic *Metallibacterium* sp. X11 and *M*. *scheffleri* (Figure 5, Table 2). These organisms, and the closely related strains A4F5, WJ2, and YE3-D1-10-CH, represent a metabolically versatile group of acidophilic, heterotrophic organisms, capable of aerobic organotrophic and lithotrophic (using Fe(II) and reduced sulfur species) metabolism, as well as anaerobic Fe(III) respiration (Coupland and Johnson, 2008; Lu et al., 2010; Delavat et al., 2012; Wu et al., 2013; Ziegler et al., 2013). This metabolic versatility [most notably the ability to oxidize Fe(II) and reduce Fe(III)] may explain the relatively uniform distribution of these phylotypes within the MF iron mound, and the apparent sustained microbial metabolism (as indicated by cell abundances; Figure 1E), regardless of the prevailing geochemical conditions. This may also explain the relatively high abundance of culturable organotrophic FeRB in regions of the iron mound that contained high DO concentrations (Figure 1).

The abundance of Cyanobacteria-affiliated phylotypes in the top 3 cm of the MF iron mound was initially surprising (Figure 4), since cyanobacteria are not frequently encountered in AMD-impacted settings (González-Toril et al., 2003), but more detailed examination of these sequences revealed that they were attributable to chloroplast 16S rRNA gene sequences of phototrophic microeukaryotes (Figure 5, Table 2). A variety of phototrophic microeukaryotes may be present in AMD-impacted setting (Rowe et al., 2007; Sánchez España et al., 2007; Senko et al., 2011), including *Euglena mutabilis* (Brake et al., 2004; Aguilera et al., 2007; Brake and Hasiotis, 2010), which was the most abundant phototrophic microeukaryotic phylotype detected in the sediments (Figure 5, Table 2). Notably, the abundance of phototrophic microeukaryotic phylotypes was coincident with relatively high DO concentration (Figures 1, 4). While it is unclear whether these organisms were active below the sediment water interface, it appears that their activities contributed to the relatively high TOC of the shallower regions of the iron mound (Figure 1D). It is not clear how light is attenuated within iron mound sediments, so we cannot determine whether the phototrophic microeukaryotes detected in deeper sediments were active, or if the DNA recovered was a remnant of organisms that were previously active near the sediment-AMD interface and were simply buried, now-inactive cells. Below 2 cm the relative abundances of phototrophic microeukaryotic phylotypes diminished dramatically (Figure 4), concurrently with the diminished abundance of TOC (Figure 1D), indicating that after burial in Fe(III) (hydr)oxides, the activities of these organisms were diminished and the organic carbon that they produced was degraded.

The most abundant Betaproteobacteria-affiliated OTU_0.03_ were taxonomically assigned by the RDP-II classifier function as *Gallionella* spp., which are neutrophilic, aerobic FeOB (Emerson et al., 2010). However, analysis of these OTU_0.03_ using BLASTn revealed that they were attributable to Fe(II) oxidizing “*Ferrovum*” spp. and *Leptothrix ocracea* (Figure 5, Table 2). “*Ferrovum*” spp. are aerobic, acidophilic, and autotrophic FeOB (Hedrich et al., 2009; Heinzel et al., 2009; Tischler et al., 2013) that have been illustrated to be the predominant organisms present in AMD-impacted systems exhibiting similar physicochemical characteristics to the MF iron mound (Hallberg et al., 2006; Brown et al., 2011). *L. ocracea* is a neutrophilic FeOB (Fleming et al., 2011), though phylotypes similar to the one recovered from the MF iron mound have been observed in AMD-impacted systems, suggesting that the phylotypes detected here may be acidophilic lineages of *Leptothrix*.

While Chloroflexi-affiliated and unassignable Bacterial phylotypes were relatively abundant in libraries from each depth interval, they increased in relative abundance in the deeper regions of the iron mound (Figure 4). Robust taxonomic assignments could not be made to the most abundant Chloroflexi-affiliated phylotypes, but phylotypes affiliated with this phylum have been detected in other AMD-impacted systems, including a physicochemically similar iron mound (Senko et al., 2008; Lucheta et al., 2013). The role of Chloroflexi in AMD-impacted systems remain unclear, but their slightly increased relative abundance in deeper portions of the iron mound suggests that they may be capable of anaerobic metabolism. Several of the unassignable Bacteria-affiliated phylotypes were 97–99% similar to sequences associated with AMD-impacted systems (Figure 5, Table 2). The prominent representation of unassignable Bacteria-affiliated sequences in libraries recovered from the deeper, anoxic portions of the iron mound suggests a large uncharacterized pool of microorganisms in anoxic portions of AMD-impacted systems in comparison to relatively well characterized near-surface portions (e.g., Senko et al., 2008; Amaral-Zettler et al., 2011; Brown et al., 2011).

Despite the high relative abundance of Gammaproteobacteria-affiliated phylotypes throughout the MF iron mound, phylotypes attributable to the well-characterized genus of acidophilic FeOB *Acidithiobacillus* did not comprise a large fraction of Gammaproteobacterial sequences. However, phylotypes attributable to *Acidithiobacillus* spp. were detected at relative abundances of 1.3–2.2% in sediments below 4 cm (Figure 5, Table 2). *Acidithiobacillus* spp. are facultatively anaerobic, capable of coupling oxidation of H_2_ or reduced sulfur species to the reduction of Fe(III) (Hedrich et al., 2011), so the higher relative abundances of *Acidithiobacillus*-phylotypes in deeper portions of the iron mound may be a reflection of their ability to metabolize under O_2_-limited conditions. Similarly, Euryarchaeota- and Nitrospirae-affiliated phyltypes attributable to the class Thermoplasmata and genus *Leptospirillum*, respectively, were most abundant in sequence libraries derived from sediments below 4 cm (Figures 4, 5). Thermoplasmata include the extremely acidophilic (optimal pH approximately 1.5), obligately aerobic autotrophic Fe(II) oxidizing *Ferroplasma* spp. (Edwards et al., 2000; Golyshina et al., 2000 science), though RDP-II-assigned taxonomy indicated that the abundant Euryarchaeal phylotypes (Figure 5) were similar to those detected in methanogenic marine sediments, suggesting the possibility of methanogenesis in anoxic regions of the iron mound sediments. *Leptospirillum* spp. are also aerobic autotrophic FeOB that are generally encountered in systems with pH <3 (Hallberg and Johnson, 2003; Tyson et al., 2005). The detection of these phylotypes in the anoxic regions of the iron mound (Figure 1B) is surprising, particularly the phylotypes attributable to *Leptospirillum* spp., which are obligately aerobic (Fujimura et al., 2012).

No clear patterns in the relative abundances of WPS-2-, AD3-, Acidobacteria, and Firmicutes-affiliated phylotypes in sequence libraries from various depths within the MF iron mound were observed, and representatives of these phyla comprised 1–5% of total sequences within the respective libraries (Figure 4). While the most abundant WPS-2-affiliated OTU_0.03_ (Figure 5) were similar to phylotypes detected in AMD-impacted systems, they could not be reliably assigned to organisms represented in cultures (Table 2). The AD3-affiliated phylotype was 99% similar to A10G4 (Figure 5, Table 2) from an acidophilic Fe(II) oxidizing enrichment culture (Wu et al., 2013). The most abundant Acidobacterial phylotype detected in the iron mound sediments was 99% similar to sequences detected in an AMD impacted system, and 97% similar to an aerobic acidophlic organotrophic organism CH1 (Diaby et al., 2007), though it is unclear if this organism might be capable of anaerobic metabolism. More detailed taxonomic analysis of Firmicutes-affiliated phylotypes revealed shifts in the evolutionary lineages to which they could be assigned (Figure 5, Table 2). In the upper 4 cm of the iron mound, Firmicutes-affiliated phylotypes were similar to the acidophilic FeOB iFeo-D4-31-CH (Figure 5, Table 2) that was isolated from an AMD-impacted lake sediments and is also capable of Fe(III) reduction (Lu et al., 2010). The isolate iFeo-D4-31-CH and several other isolates from the same system were attributable to *Alicyclobacillus* spp., as was OTU_0.03_ 32, which was abundant throughout the depth of the MF iron mound (Figure 5, Table 2). An OTU_0.03_ attributable to the acid-tolerant sulfate- and Fe(III)-reducing bacterium *Desulfosporosinus* GBSRB4.2, which was isolated from iron mound sediments (Senko et al., 2009), was detected in the lower 2 cm of the iron mound sediments (Figure 5, Table 2).

### Biogeochemical processes associated with MF iron mound sediments

Geochemical characterization of iron mound sediments indicated redox cycling of Fe was occurring in the MF iron mound, and microbiological characterization of the iron mound system revealed communities capable of Fe metabolism regardless of depth. At the MF, vertical growth of the iron mound [by Fe(III) (hydr)oxide deposition] is mediated by the activities of aerobic FeOB near the sediment-AMD interface, supported by O_2_ produced by phototrophic microeukaryotes and diffusing into the AMD from the atmosphere. Organic carbon appears to be derived from phototrophic microeukaryotic activities, and perhaps autotrophic FeOB activities near the iron mound-AMD interface. With progressively greater depth, we observed depletion of O_2_, and FeRB activities gave rise to a peak in porewater Fe(II) concentration at approximately 2.5 cm below the sediment-AMD inferface (Figure 1A). This region of the iron mound is also where the transformation of poorly crystalline Fe(III) phases to goethite occurred (Figure 2), which was likely induced by FeRB activities (Hansel et al., 2003; Burton et al., 2007; Bertel et al., 2012). However, it is notable that the region of maximal Fe(III) reduction [as indicated by maximal porewater Fe(II) concentration] was not completely depleted in O_2_, and culturable FeRB were most abundant in the upper regions of the iron mound (Figures 1A,B,D). This indicates a co-occurrence of both O_2_ and Fe(III) reduction within the iron mound sediments. Thus far, no obligately anaerobic and Fe(III)-respiring bacteria have been isolated from AMD-impacted systems. Those that have been recovered in culture are facultative anaerobes (Johnson and McGinness, 1991; Pronk et al., 1992; Küsel et al., 1999). Indeed, acidophilic, organotrophic *Acidiphilium* spp. exhibit more robust Fe(III)-reducing activities under microaerobic conditions rather than under strictly anaerobic conditions (Küsel et al., 2002; Malki et al., 2008), and close association of FeOB and FeRB activities has been recently reported in circumneutral settings (Elliott et al., 2014).

Characterization of microbial communities at various depths within the iron mound sediments indicated transitions within the microbial communities with depth, with a demarcation between the communities above and below 4 cm (Figure 3), and phylotypes attributable to lineages of anaerobic microorganisms below this point (Figure 5). However, culturable organotrophic FeRB abundances were highest at the 2 cm depth interval and decreased with depth (Figure 1E), while dissolved Fe(II) was depleted between 3 and 5 cm (and remained relatively low below 5 cm; Figure 1A). Similarly, the most dramatic decrease in sulfate concentration occurred at the sediment-AMD interface [likely due to incorporation of sulfate into biogenic Fe(III) phases], and no sulfate depletion was evident in deeper regions of the sediments, suggesting minimal sulfate reducing bacterial (SRB) activity (Figure 1C). No black FeS phases were observed in the sediment cores, and FeS phases (e.g., mackinawite or greigite) could not be detected by XRD, but it is likely that sulfide accumulation in the sediments would be minimal due to rapid oxidation of biogenic sulfide by Fe(III) phases in the iron mound sediments (dos Santos Afonso and Stumm, 1992). The organic carbon content of the sediments below 4 cm (approximately 2 mg/g sediment; 0.1 mmol CH_2_O/g) is sufficient to support the reductive dissolution of Fe(III), yielding over 100 mM Fe(II) in porewater, and at relatively low pH, the thermodynamic favorability of goethite-Fe(III) reduction would likely be enhanced (Bethke et al., 2011). Taken together, these results indicate less anaerobic respiratory activity [i.e., Fe(III) and sulfate reduction] in the MF sediments below approximately 3 cm, despite depletion of O_2_ below this point.

As such, the demarcation between aerobic and anaerobic communities within the iron mound sediments appears to be somewhat nebulous. Total cell abundances were uniform throughout the iron mound sediments (Figure 1E), despite minimal evidence of anaerobic respiratory activities in the deeper, O_2_-depleted portions of the sediments. Similarly, relatively uniform abundances of culturable FeOB (Figure 1E) were detected throughout the iron mound (Figure 1E). The most abundant phylotype detected in the iron mound (comprising ≥20% of sequences, regardless of depth) was attributable to a lineage of acidophilic bacteria capable of aerobic Fe(II) oxidation and anaerobic Fe(III) reduction, and phylotypes attributable to aerobic FeOB *Leptospirillum* spp. and *Acidithiobacillus* spp. were most abundant in sequence libraries derived from iron mound sediments at depth intervals below 3 cm (Figure 5, Table 2). Aqueous Fe(II) was depleted below the region of complete O_2_ depletion, and remained at a relatively low concentration in deeper regions of the iron mound, despite sufficient organic carbon to support abundant Fe(III) reduction (Figures 1A,B,D). Removal of aqueous Fe(II) by adsorption is an unlikely mechanism of dissolved Fe(II) depletion at depths below 3 cm, because solid associated Fe(II) was similarly depleted at depth within the iron mound sediments (Figure 1A). As such, it appears that the depletion of dissolved Fe(II) and maintenance of relatively low Fe(II) concentrations is attributable to Fe(II) oxidation. A similar pattern of Fe(II) oxidation at depth has been observed in a similar system, but in that case, Fe(II) oxidation was supported by O_2_ delivered by interstitial water flow (Larson et al., 2013). However, in the case presented here, O_2_ was not detected in deeper sediments where Fe(II) oxidation appeared to occur, so it is unclear how this might be accomplished in the absence of light or chemical oxidants (e.g., nitrate or O_2_). Oxidation of Fe(II) in O_2_-depleted portions of the iron mound may be facilitated by extracellular electron transfer processes to oxic portions of the iron mound (Ntarlagiannis et al., 2009; Nielsen et al., 2010; Roden et al., 2010; Kato et al., 2012; Risgaard-Petersen et al., 2012), and such processes will be further explored in iron mound settings. This work illustrate that while the initial development and upward growth of the iron mound is mediated by FeOB with ready access to O_2_, Fe(III) bioreduction also occurs in oxic portions of the iron mound, but Fe(II) oxidation can be sustained at O_2_-depleted depths within the sediments.

### Conflict of interest statement

The authors declare that the research was conducted in the absence of any commercial or financial relationships that could be construed as a potential conflict of interest.
